# The relationship between occupant behaviour and indoor air quality in Malaysian hospital outpatient departments: A multistage cross-sectional study

**DOI:** 10.1016/j.heliyon.2024.e34454

**Published:** 2024-07-10

**Authors:** Farha Ibrahim, Ely Zarina Samsudin, Ahmad Razali Ishak, Jeyanthini Sathasivam

**Affiliations:** a Department of Public Health Medicine, Faculty of Medicine, Universiti Teknologi MARA, Sungai Buloh, Selangor, Malaysia; b Training Management Division, Ministry of Health, Putrajaya, Malaysia; c Centre for Environmental Health and Safety, Faculty of Health Sciences, Universiti Teknologi MARA, Puncak Alam, Selangor, Malaysia; d Public Health Division, Johor State Health Department, Ministry of Health, Johor Bahru, Malaysia

**Keywords:** Indoor air quality, Occupant behaviour, Occupant practice, Occupant factors, Hospital, Healthcare

## Abstract

**Introduction:**

Poor indoor air quality (IAQ) in healthcare settings may adversely impact occupants’ well-being and promote transmission of infectious respiratory disease. However, evidence on its potentially modifiable determinants, including occupant behaviour, remains scarce. This study aims to determine the relationship between occupant behaviour and IAQ in Malaysian hospital outpatient departments (OPDs).

**Methods:**

A multistage cross-sectional study of six randomly selected Malaysian public hospital OPDs was conducted. In stage one, IAQ parameters, including temperature, relative humidity (RH), air velocity (AV), carbon dioxide (CO_2_), total bacterial count (TBC), and total fungal count (TFC) were measured. In stage two, an observation form based on the Korsavi and Montazami tool for measuring adaptive behaviour was used to examine occupant density, activities, and operation of building envelopes and appliances. Simple correlation, partial correlation, and linear regression analyses were performed to examine the relationship between occupant behaviour and IAQ parameters.

**Results:**

The IAQ of selected hospital OPDs complied with established standards, except for temperature and AV. Occupant density was positively correlated with temperature and CO_2_. Meanwhile, occupants' activities including slow walking and brisk walking were positively correlated with temperature, AV, CO_2_, TBC and TFC. Conversely, occupants' opening of windows and doors were positively correlated with temperature and AV but negatively correlated with CO_2_, TBC and TFC. Finally, turning on fans was positively correlated with AV but negatively correlated with TBC, whereas turning on air conditioner was positively correlated with CO_2_. Among occupants’ behaviour, opening of windows and doors contributed the most to variation in IAQ parameters.

**Conclusions:**

The study findings suggest that IAQ in hospital OPDs are influenced by occupant density, activities, and operation of doors, windows, and appliances. Prospective hospital IAQ guidelines should incorporate policies and measures targeting these factors to ensure occupants’ best practices in maintaining healthy hospital indoor air environments.

## 1 Introduction

Indoor air quality (IAQ) refers to the air quality within and around buildings and structures, especially concerning the health and comfort of building occupants [1]. Good IAQ is essential to ensure occupants' health and well-being, as deterioration in IAQ may lead to adverse health effects and hamper occupants’ cognitive function, productivity, and efficiency [2,3]. In specialised settings such as healthcare facilities, the indoor air environment may also contain contaminants in the form of bioaerosols, such as fungal spores, bacterial cells, viruses, and pollen grains [[4], [5], [6], [7]]. Poor IAQ resulting from inadequate ventilation and high levels of indoor air biological contaminants in these settings may lead to building-related illnesses such as allergic reactions and sinusitis, as well as the transmission of infectious respiratory diseases such as SARS-CoV-2 infection and influenza-like illness [8,9]. Indeed, infectious bioaerosols present within hospital indoor air have been linked to various hospital-acquired infections (HAIs) [10,11], and indoor air temperature, relative humidity (RH), and carbon dioxide (CO_2_) levels have been shown to influence the spread of infectious diseases by promoting indoor pathogen survival and transmission [12]. Such person-to-person transmission of infectious agents through recirculated indoor air may lead to significant morbidity and mortality for hospital building occupants [13].

There are multiple contributing factors for IAQ in hospital settings, as hospital indoor environments are complex ecosystems influenced by building design, operation, and maintenance, as well as numerous interactions between the indoor environment and its occupants [12,14]. One of the factors significantly impacting IAQ is occupant behaviour, particularly in terms of bioaerosol concentrations [[15], [16], [17], [18], [19]]. Occupant behaviour is a comprehensive reflection of occupants' preferences and habits in a building, including their presence, movements and actions [20]. It is generated by the comprehensive effect of both ‘sensory stimuli’ and ‘non-sensory stimuli’ towards the occupants, where adjustments in occupant behaviour can be summarized as “occupants processing the information obtained from the surrounding environment and giving certain feedback accordingly” [20,21]. In healthcare facilities, occupant behaviour includes clinical activities, therapies, and operation of medical equipment [22], as well as occupants' activities, movement, time spent in a building, and operation of building envelopes and appliances [23].

Evidence suggests that occupant activities in hospitals contribute to the generation of indoor air contaminants such as particulate matter (PM), chemical pollutants including CO_2_, and bioaerosols [23]. In this regard, indoor bioaerosols produced by occupants have been shown to vary significantly according to their activities inside the room, such as sitting, standing, or moving around [24]. Activities such as talking, sneezing, coughing, walking, washing, and flushing the toilet may also produce indoor biological pollutants [22,25]. In addition, building occupant density and occupant activity intensity have been reported to influence the concentration of CO_2_ in a building [26], where a high CO_2_ level was associated with a higher probability of spread of infections in indoor spaces [27]. In fact, previous studies have indicated that the likelihood of indoor airborne disease transmission can be estimated using continuous CO_2_ measurements [28].

In addition to occupant density and occupants' activities, hospital IAQ may also be influenced by occupants' adaptive behaviour, which is performed in response to their perception of the indoor environment. These behaviours include adjusting the ventilation system, utilising air-conditioning units and fans, and the opening and closing of windows and doors [29]. In this regard, the usual practice of doors and windows being left open to enable fresh air to flow in during cooler weather and doors and windows being closed and air conditioner (AC) utilised during hotter weather may cause IAQ-related issues [30]. This is because opening windows and doors may expose occupants to outdoor air pollutants [31], whereas utilising AC units in enclosed environments may result in high indoor CO_2_ concentrations, both of which impact occupants’ health [32]. Indeed, Chen et al. [30] compared the IAQ in enclosed air-conditioned classrooms with fresh air ventilation classrooms and found that enclosed air-conditioned classrooms tend to have substandard CO_2_ and PM_2.5_. On the other hand, studies have shown that opening windows may lead to a rise in pollutants from outdoor sources such as nitrogen dioxide and PM_2.5_ [33,34].

Given the significant impact of occupants' behaviour on IAQ and the negative repercussions of poor IAQ on occupants' health, a greater appreciation of the effect of occupants' behaviour on IAQ, specifically in hospital settings, is warranted. However, despite the attention placed on strategies to limit infection risks in specialised areas within healthcare facilities such as the operating room and wards in previous years [35], areas such as the outpatient department (OPD), where the crowd is most common, are still neglected [36,37]. The OPD is one of the most visited hospital departments and from 1990 to 2016, the outpatient volume had been reported to increase from 24.8 billion to 39.35 billion visits globally [38]. The increasing utilisation of outpatient healthcare has led to congested and crowded environments in the OPD areas [39], which is especially concerning in terms of disease transmission as most OPD visitors tend to be those at higher risk of getting infections [9,40]. In fact, a previous review had suggested that given the sizable number of infection cases reported in outpatient healthcare settings, in-patient infection control measures should be extended to these settings [41]. In addition, evidence on factors influencing IAQ in healthcare settings remains insufficient compared to other building types [42], especially pertaining to occupant-related factors [14]. Therefore, this study aims to investigate the relationship between occupant behaviour and IAQ in the OPD of Malaysian hospitals. An improved understanding of the influence of occupant behaviour on hospital IAQ would enable practical recommendations for occupants’ best practices to promote good IAQ and minimize the transmission of infectious diseases within healthcare facilities [5].

## 2 Materials and methods

### 2.1 Study design, setting, and population

This is a multistage cross-sectional study conducted in Malaysian public hospitals in Johor, Malaysia. Johor is the third largest state in Peninsular Malaysia, with twelve public hospitals, including one state hospital, four major specialist hospitals, one minor specialist hospital, one psychiatric hospital, and five district hospitals, serving a population of 3.8 million people [43,44]. To select the study sites, stratified random sampling was performed. First, the Johor public hospital OPDs were classed according to the age of the building. The rationale for this was that the building age has been found to significantly influence IAQ [45]. Hospital OPD buildings aged 15 years or older were considered old OPD buildings, while those aged less than 15 years were considered new OPD buildings [46]. Simple random sampling was conducted to select three new and three old hospital OPDs from each stratum.

In stage one of the study, the IAQ of the six selected hospital OPDs was measured and compared against established standards. In stage two, observation of occupant behaviour in selected hospital OPDs was conducted. Occupants included were healthcare workers (HCWs) comprising of medical officers, staff nurses, community nurses, assistant medical officers, and other administrative staff assigned to work in the hospital OPD during the data collection period. Individuals working in locations outside the designated OPD area and those not specifically assigned to work within the selected OPDs were excluded from the study.

**Fig. 1:**
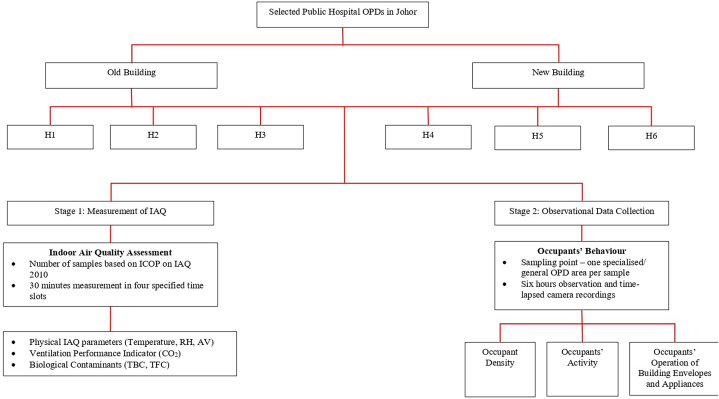
Study design, sampling, and flow.

Prior to data collection, ethical approval from the UiTM Research Ethics Committee [REC/08/2022 (PG/MR/193)], Medical Research Ethics Committee [NMRR ID-22-00842-VCH (IIR)], and permission to conduct the study from respective hospital directors were obtained. Written informed consent was obtained from all participants. Initial data collection included retrieving data regarding the geographical location of the hospital (urban/rural), size of OPD area, total number of doors, windows, AC, and fans within the OPD area (including those in consultation rooms, waiting areas, and procedure rooms), material composition of the OPD, type of ventilation system, environmental control measures (if any), maintenance, hygiene, and surveillance services for the ventilation system and appliances, and renovation history from the Engineering Division of Johor Health State Department. Subsequently, data retrieved were verified through site visits at each study sites to ensure that any latest changes made in the hospital OPD were noted. The study was conducted from September 26, 2022 until December 29, 2022, and the study design, sampling, and flow is summarised in Fig. 1.

### 2.2 Stage one: IAQ measurement in selected hospital OPDs

The evaluation of IAQ was carried out in accordance with the Industry Code of Practice (ICOP) on IAQ 2010 published by the Department of Occupational Safety and Health (DOSH) Malaysia [47]. In this study, an intermittent measurement strategy was employed, averaging half-hour measurements conducted during four specified time slots. The timeslots were chosen based on the building operation pattern and were distributed evenly over the OPD operating hours, covering the period of highest OPD occupancy. *A* *priori* number of sampling points in each hospital OPD was determined according to the recommended minimum number of sampling points for IAQ assessment outlined both in the ICOP [47] and Guideline on IAQ for Hospital Support Service [4]. The sampling done from the first sampling point to the N^th^ sampling point in the first sampling session was repeated in the same sequence for the subsequent 3 sampling sessions [4]. To account for temporal variation due to variations that may occur in different time of the day and day of the week, the time slot and day of the week of each IAQ measurements were recorded. Temporal variation due to seasonal change was not considered as Malaysia has a tropical climate, with minimal seasonal variation in average monthly temperature, fluctuating only by 1 °C [48].

The assessed indoor air parameters comprised of three physical IAQ parameter including temperature, relative humidity (RH), and air velocity (AV), ventilation performance indicator, CO_2_, and two biological contaminant parameters, i.e., total bacterial count (TBC) and total fungal count (TFC). Three air monitoring instruments including the TSI Quest EVM Environmental Monitor, the UT363S Digital Anemometer, and the Merck microbiological monitoring air sampler, M Air T were utilised. The TSI Quest EVM Environmental Monitor was employed to measure indoor air temperature, RH, and CO_2_, whereas the UT363S Digital Anemometer was used to measure AV.

An active sampling technique was utilised to collect samples for TBC and TFC using the M Air T sampler. Two types of agar plates, specifically malt extract agar for TBC and tryptone soy agar for TFC, were employed as the agar medium. These plates were positioned inside the M Air T sampler, which was configured with an initial airflow rate of 140 L/min for the first 500 L, followed by 180 L/min until reaching a total air sampling of 1000 L. Subsequently, the agar plates were then transported to the laboratory for incubation and analysis of colony growth within 24 hours of the sampling process. Tryptone soy agar plates for TBC analysis were tested by using In House Method, incubated at 35 °C for 48 hours [49], while malt extract agar plates for TFC analysis were tested by using In House Method, incubated at 25 °C for 5 days [50]. The growth of all colonies in both malt extract agar and tryptone soy agar plates were promptly counted after the incubation period was completed, which is 48 hours ± 2 hours for tryptone soy agar, and 5 days ± 2 hours for malt extract agar, respectively. Colony count was then recorded as colony forming unit (cfu) per plate [51], which is equivalent to cfu per meter cube (cfu/m^3^) as the amount of air sample volume impacted onto the agar plate is equivalent to 1000 L (1 m^3^). Uninoculated sterile media plate served as the negative control. Samples of sterility (negative control) for each lot of samples were also recorded [51].

All indoor air measurement results were then analysed and compared to the standard guidelines established in the ICOP on IAQ 2010 by DOSH Malaysia [47] and the American Society of Heating, Refrigerating and Air-Conditioning Engineers (ASHRAE) standard 62.1 2022, ASHRAE standard 55 2017, and ASHRAE standard 170 2021 [[52], [53], [54]]. These standards were chosen as the ICOP is commonly used as a local standard for IAQ in Malaysia [[55], [56], [57], [58]] whereas the ASHRAE standards are well-known international standard guidelines for IAQ in various settings, including healthcare facilities [52].

### 2.3 Stage two: occupant behaviour assessment in selected hospital OPDs

Occupant behaviour was evaluated using an observation form adapted from Korsavi and Montazami [59]. The form comprised 12 items assessing three aspects: occupancy, occupants' activities, and occupants’ operation of building envelopes and appliances. According to the authors, the consistency and reliability of the form were indicated by the low standard deviations clustering around the mean of findings, and the low percentage of inconsistent data (<1 %) implies the validity of responses and the applied methodology.

*A priori* sample size calculation based on the correlation between the width of windows opened and AV identified from a previous study by Howard-Reed et al. [60], with α set at 0.05 and β set at 0.2, was determined to be 14, using the online calculator for correlation sample size by Hulley, Cummings [61].

An observation was conducted in every specialty clinic OPD area in hospitals with in-house medical specialists and in the general OPD area in hospitals without in-house medical specialists. . Each observation session lasted 6 hours, which was deemed sufficient and captured at least 60 % of the occupant behaviour and operation in the hospital OPD during the 8.00 a.m. to 5.00 p.m. working hours. Additionally, time-lapse camera recordings were made during each observation at every site to validate the findings from the observation forms.

Usage intensity, i.e., occupancy in the selected OPDs by HCWs, patients, and visitors, was calculated based on the occupant density per 100 square meters [62]. The total number of occupants was counted, and the area of the space was then measured. The occupant density was then calculated using the formula [62]:

$$\begin{array}{c} Occupant\;density\;per\;100\;m^{2}=\frac{number\;of\;occupants}{area\;of\;the\;space}\times 100 \end{array}$$

The occupants' activities were recorded as the average number of hours spent engaging in activities varying from light to high intensity. These activities encompassed sitting, standing, slow walking, brisk walking, pushing loaded trolleys, and lifting heavy weights. Meanwhile, the operation of building envelopes and appliances by the occupants were documented, which include the percentage of doors and windows opened, the average percentage of fans and AC in use, and the average frequency of adjustments made by occupants to doors, windows, AC temperature settings, and fan speed settings. To provide context for OPD occupants’ behaviour, the weather conditions and time of the day were recorded.

### 2.4 Statistical analysis

Statistical analyses were performed using IBM Statistical Package for the Social Sciences version 28.0. Initial data analysis included assessment of missingness and influential data, as well as model checking for correlation and regression analyses. Descriptive analysis included frequency and percentage for categorical data and mean and standard deviation for numerical data. Simple and partial correlations were then performed to examine the correlation between occupant behaviour and IAQ in the selected hospital OPDs. First, simple correlation analysis was conducted to examine the bivariate correlation between occupants' behaviour and IAQ parameters. Subsequently, partial correlation analysis was performed to enable adjustment for potential confounders identified from the literature, including where data is available, the age of the building [63], geographical location of the hospital [64], OPD area size [65], occupant density [66], ventilation system and appliances maintenance, hygiene, surveillance, and renovation history [66], type of ventilation system [67], environmental control measures (if any) [68], material composition of the OPD [14], and covariates significant at a p value of 0.25 identified from the simple correlation analyses. The adjusted correlation coefficient was reported as the correlation coefficient, r, where an r with a p value of less than 0.05 was considered to be statistically significant. Based on Cohen's d [69], the effect sizes were reported as follows: correlations ranging from 0.10 to 0.29 as weak, correlations from 0.30 to 0.49 as moderate, and correlations from 0.50 to 0.99 as strong.

In addition, multiple linear regression was also performed to estimate the R^2^ value, which shows the proportion of the variation in the dependent variable that is explained by the predicting variable [70,71]. Similarly, the regression analyses controlled for potential confounders identified from the literature, including where data is available, the age of the building [63], geographical location of the hospital [64], OPD area size [65], occupant density [66], ventilation system and appliances maintenance, hygiene, surveillance, and renovation history [66], type of ventilation system [67], environmental control measures (if any) [68], material composition of the OPD [14], and covariates significant at a p value of 0.25 identified from the simple linear regression analyses. All factors underwent an interaction check to ascertain their influence on IAQ parameters, specifically to identify any synergistic or antagonistic effects [72]. Factors with significant interactions were included in the final logistic regression model.

To examine the robustness of the study findings, sensitivity analyses were performed to test the stability of the results under different assumptions and model specifications. The primary model (Model 1) included all study data, Model 2 included all study data as well as the variable measurement period according to time slot of the day, and Model 3 included all study data as well as the variable measurement period according to day of the week. The differences in partial correlation coefficients and R^2^ values were compared with the primary analysis.

## 3 Results

### 3.1 Characteristics of selected hospital OPDs and occupants

All six selected public hospital OPDs were located in urban locations. The age of the hospital OPD buildings ranged from 28 to 110 years and 11 to14 years for old OPDs and new OPDs buildings, respectively. Two hospital OPDs were mechanically ventilated, while the other four were naturally ventilated. The same concession company served all six hospitals and provided mechanical ventilation air conditioning (MVAC) and appliance hygiene and maintenance services outlined in the public hospitals’ service agreement. Concurrently, the engineering division officers from the state health department conducted surveillance for MVAC and appliances in the public hospital OPDs. Thus, the frequency of hygiene, maintenance, and surveillance activities for all selected public hospital OPDs were identical based on the ventilation system they were equipped with. According to the officers, no structural renovation had been conducted in the selected OPDs, and no additional environmental control measures such as the use of air purifiers were implemented in the selected OPDs. Meanwhile, the information on the material composition of the selected OPDs were not available from the hospital records of the hospital engineering division.

A total of 151 HCWs working in the six selected public hospital OPDs during data collection were observed in this study, involving 32 observation sites. They comprised medical specialists (in-house or visiting), medical officers, medical assistants, nurses, healthcare assistants, and clerks. Within the 6 hours of observation, HCWs remained within the OPD area but constantly moved from one place to another. A summary of the characteristics of selected hospital OPDs and occupants is outlined in Table 1.

**Table 1:** Selected hospital OPDs and occupants’ characteristics.

Hospital	Age	Category	Ventilation type	Geographical location of hospital	OPD space area	Number of measurement point	Total no. of HCWs observed, n
H1	11	New	Mechanical	Urban	2799.5 m^2^	8	18
H2	12	New	Natural	Urban	1201.2 m^2^	3	14
H3	14	New	Natural	Urban	400.3 m^2^	2	14
H4	28	Old	Mechanical	Urban	161.2 m^2^	2	8
H5	85	Old	Natural	Urban	13,714 m^2^	15	90
H6	110	Old	Natural	Urban	218.6 m^2^	2	7

### 3.2 IAQ of selected hospital OPDs and comparison with IAQ standards

The IAQ of selected hospital OPDs is summarised in Table 2. Based on the comparison of IAQ findings with the ICOP on IAQ 2010 and ASHRAE standards, the mean IAQ parameters in the selected public hospital OPDs were within the normal range except for the temperature and AV. For temperature, it was observed that hospital OPDs with natural ventilation tended to have indoor temperatures above ICOP and ASHRAE standards. Meanwhile, mechanically ventilated OPDs tended to have indoor temperatures below standard limits. In relation to AV, OPDs with natural ventilation tended to have AV above the ICOP but within ASHRAE recommended levels, while mechanically ventilated OPDs tended to have AV below the ICOP recommended level.

**Table 2:** The summary of IAQ parameter in the selected public hospital OPDs and its comparison with the standards guidelines.

Variables		H1	*ICOP 2010*	*ASHRAE*	H2	*ICOP 2010*	*ASHRAE*	H3	*ICOP 2010*	*ASHRAE*	H4	*ICOP 2010*	*ASHRAE*	H5	*ICOP 2010*	*ASHRAE*	H6	*ICOP 2010*	*ASHRAE*
**Temp (*°C)***	Min	**20.28**	***X***	***X***	**22.91**	***X***	***X***	**28.05**	***X***	***X***	**22.68**	***X***	***X***	**27.59**	***X***	***X***	**27.97**	***X***	***X***
	Max	**21.89**	***X***	***X***	25.80	*✓*	*✓*	**29.30**	***X***	***X***	24.00	*✓*	*✓*	**29.10**	***X***	***X***	**28.70**	***X***	***X***
	Mean (SD)	**21.00 (**0.87)	***X***	***X***	24.80 (2.25)	*✓*	*✓*	**28.60** (0.49)	***X***	***X***	23.40 (0.46)	*✓*	*✓*	**28.30** (1.10)	***X***	***X***	**28.00** (0.27)	***X***	***X***
**RH (%)**	Min	55.20	*✓*	*✓*	40.50	*✓*	*✓*	60.90	*✓*	*✓*	62.60	*✓*	*✓*	55.80	*✓*	*✓*	60.10	*✓*	*✓*
	Max	**73.40**	***X***	***X***	**74.10**	***X***	***X***	**68.88**	*✓*	***X***	67.10	*✓*	**X**	**70.90**	***X***	***X***	65.00	*✓*	**X**
	Mean (SD)	62.68 (4.02)	*✓*	*✓*	54.40 (11.11)	*✓*	*✓*	64.70 (2.63)	*✓*	*✓*	64.11 (1.25)	*✓*	*✓*	63.43 (3.98)	*✓*	*✓*	62.70 (1.61)	*✓*	*✓*
**AV (m/s)**	Min	**0.12**	***X***	*✓*	**0.12**	***X***	*✓*	**0.67**	***X***	*✓*	**0.12**	***X***	*✓*	0.43	*✓*	*✓*	0.45	*✓*	*✓*
	Max	0.16	*✓*	*✓*	**0.74**	***X***	*✓*	**0.94**	***X***	*✓*	0.16	*✓*	*✓*	**0.94**	***X***	*✓*	**0.67**	***X***	*✓*
	Mean (SD)	**0.14** (0.02)	***X***	*✓*	0.30 (0.24)	*✓*	*✓*	**0.79** (0.07)	***X***	*✓*	**0.14** (0.02)	***X***	*✓*	**0.62** (0.11)	***X***	*✓*	**0.55** (0.05)	***X***	*✓*
**CO_2_ (ppm)**	Min	411.00	*✓*	*✓*	328.00	*✓*	*✓*	642.00	*✓*	*✓*	855.00	*✓*	*✓*	415.00	*✓*	*✓*	360.00	*✓*	*✓*
	Max	822.00	*✓*	*✓*	683.00	*✓*	*✓*	**1097.00**	***X***	***X***	988.00	*✓*	*✓*	**1170.00**	***X***	***X***	663.00	*✓*	*✓*
	Mean (SD)	566.13 (103.88)	*✓*	*✓*	485.92 (93.09)	*✓*	*✓*	848.04 (121.05)	*✓*	*✓*	946.50 (45.36)	*✓*	*✓*	687.63 (221.40)	*✓*	*✓*	499.08 (96.44)	*✓*	*✓*
**TBC (cfu/m**^**3**^**)**	Min	11.75	*✓*	*N/A*	9.33	*✓*	*N/A*	63.00	*✓*	*N/A*	38.50	*✓*	*N/A*	45.00	*✓*	*N/A*	5.00	*✓*	*N/A*
	Max	32.64	*✓*	*N/A*	21.00	*✓*	*N/A*	94.00	*✓*	*N/A*	70.00	*✓*	*N/A*	86.26	*✓*	*N/A*	23.00	*✓*	*N/A*
	Mean (SD)	21.85 (10.50)	*✓*	*N/A*	13.46 (7.20)	*✓*	*N/A*	74.10 (0.10)	*✓*	*N/A*	53.41 (13.08)	*✓*	*N/A*	62.37 (14.72)	*✓*	*N/A*	16.16 (7.98)	*✓*	*N/A*
**TFC (cfu/m**^**3**^**)**	Min	4.50	*✓*	*N/A*	3.66	*✓*	*N/A*	15.00	*✓*	*N/A*	12.00	*✓*	*N/A*	15.40	*✓*	*N/A*	6.00	*✓*	*N/A*
	Max	14.50	*✓*	*N/A*	7.33	*✓*	*N/A*	28.00	*✓*	*N/A*	20.50	*✓*	*N/A*	25.60	*✓*	*N/A*	16.00	*✓*	*N/A*
	Mean (SD)	8.71 (6.59)	*✓*	*N/A*	5.33 (2.29)	*✓*	*N/A*	21.00 (6.66)	*✓*	*N/A*	16.50 (3.93)	*✓*	*N/A*	20.22 (9.11)	*✓*	*N/A*	8.83 (4.53)	*✓*	*N/A*

Note: Bolded values are outside of the acceptable range.

Acceptable limits: (1) ICOP 2010: Temperature (23–36 °C), RH (40–70 %), AV (0.15–0.50 m/s), CO_2_ (C1000 ppm), TBC (500 cfu/m^3^), TFC (1000 cfu/m^3^).

(2) ASHRAE: Temperature (23–36 °C), RH (<65 %), AV with airspeed control (No airspeed limit), AV with no airspeed control (T < 23 °C ≤ 0.2 m/s, T ≥ 23 °C ≤ 0.80 m/s), CO_2_ (C1000 ppm), TBC (500 cfu/m^3^), TFC (1000 cfu/m^3^).

Comparison of the IAQ parameters between old and new hospital OPD buildings (Table 3) demonstrated significant mean differences in indoor air temperature (p value < 0.001), AV (p value < 0.001), CO_2_ (p value = 0.002), TBC (p value < 0.001), and TFC (p value < 0.001).

**Table 3:** Differences in IAQ parameters of old and new selected hospital OPDs.

Indoor air parameter		t (df)	Mean difference	95 % CI		p-value
				Upper limit	Lower limit	
**Physical**	Temperature	5.40 (30)	4.66	2.89	6.42	<0.001***
	RH	1.03 (30)	2.16	−2.31	6.64	0.31
	AV	3.90 (30)	0.284	0.13	0.43	<0.001***
**Ventilation performance indicator**	CO_2_	3.03 (30)	167.53	54.71	280.35	0.002**
**Biological contaminant**	TBC	3.68 (30)	28.55	12.57	44.53	<0.001***
	TFC	3.56 (30)	8.89	3.79	14.00	<0.001***

Note: p value based on independent *t*-test. CI: Confidence interval, df: degree of freedom, t: test statistics. *Mean difference is significant at the 0.05 level (2-tailed), **Mean difference is significant at the 0.01 level (2-tailed), *** Mean difference is significant at <0.001 level (2 tailed).

### 3.3 Occupant density, activities, and operation in selected hospital OPDs

In this study, it was observed that H2 had the lowest occupant density (4.16 per 100 m^2^) while H4 had the highest occupant density (55.82 per 100 m^2^) (Table 4). According to the ASHRAE Standard [54], to achieve the minimum ventilation rates in breathing zones for outpatient facilities in healthcare settings, the default occupant density must be less than or equal to 20 persons per 100 m^2^ in various types of rooms in outpatient facilities [54]. The occupant density exceeded the default values in H3, H4, H5 and H6 (range 22–55 persons per 100 m^2^), while H1 and H2 had acceptable occupant density (range 4–5 persons per 100 m^2^).

**Table 4:** Occupant density and occupants’ activities in the selected public hospital OPDs.

Study site	Occupant density, persons per 100m^2^	Light intensity activity			Moderate intensity activity		
		Sitting, mean hours (% of observation time)	Standing, mean hours (% of observation time)	Slowly walking, mean hours (% of observation time)	Brisk walking, mean hours (% of observation time)	Pushing loaded trolley, mean hours (% of observation time)	Lifting heavy weight, mean hours (% of observation time)
H1	5.32	1.72 (28.71 %)	0.64 (10.80 %)	3.61 (60.19 %)	0 (0 %)	0.02 (0.31 %)	0 (0 %)
H2	4.16	1.04 (17.34 %)	1.20 (20.04 %)	3.75 (62.60 %)	0.06 (1.00 %)	0 (0 %)	0 (0 %)
H3	33.75	3.56 (59.33 %)	0.82 (13.69 %)	1.41 (23.61 %)	0.07 (1.17 %)	0 (0 %)	0 (0 %)
H4	55.82	2.77 (46.18 %)	0.73 (12.15 %)	2.43 (40.63 %)	0 (0 %)	0 (0 %)	0 (0 %)
H5	27.41	1.03 (17.22 %)	1.24 (20.83 %)	3.38 (56.39 %)	0 (0 %)	0.18 (2.96 %)	0.16 (2.59 %)
H6	22.93	1.63 (27.18 %)	1.13 (18.85 %)	3.23 (53.97 %)	0 (0 %)	0 (0 %)	0 (0 %)

The observation period and weather condition were 8.00 a.m. until 5.00 p.m. and sunny throughout the day for all selected hospital OPDs, respectively. Occupants were observed to mainly perform light-intensity activities throughout the 6-hour observational period, in which they were either sitting, standing, or walking slowly within the OPD area over 90 % (>5 hours) of the observation time, whereas less than 10 % of the observation time (<1 hour) was spent on moderate-intensity activities such as brisk walking, pushing a loaded trolley, or lifting heavy weights (Table 4). High-intensity activities were not performed. Among moderate-intensity activities, pushing loaded trolley were only observed in H1, a minor specialist hospital and H5, a tertiary hospital. On the other hand, lifting heavy weight was only observed in H5, a tertiary hospital. Overall, a higher variety of occupants activities were observed in tertiary hospital OPD compared to smaller hospital OPDs.

In relation to the operation of building envelopes and appliances (Table 5), the percentage of doors opened ranged from 30.8 % to 60.0 %, while the door adjustment frequency ranged from two to five times, whereas the percentage of windows opened ranged from 6.51 % to 50 %, while the window adjustment frequency ranged between two and six times. A higher percentage of doors and windows opened and higher frequency of door and window adjustment were observed in OPDs with natural ventilation compared to those with mechanical ventilation, as occupants in mechanically ventilated OPDs tended not to open or adjust their doors and windows to maintain the cool temperature indoors. In these settings, the operation of doors was mainly for entry and exit purposes by visitors and HCWs, however a small number of doors and windows located in isolated areas of the OPD such as near washrooms and areas connecting the OPD with other hospital areas were opened. In H6 (naturally ventilation OPD), specific instructions by the hospital director were given to the OPD staff since the advent of the Coronavirus Disease 2019 pandemic to open windows for approximately 5 to 10 minutes every hour in every air-conditioned room to allow adequate air exchange within the OPD area. Thus, the observed frequency of window adjustment was highest in H6.

**Table 5:** Occupants’ operations of doors, windows, and appliances in the selected public hospital OPDs.

Study site	Total no. of doors, n	Doors opened, n (%)	Doors' adjustment frequency, n	Total no. of windows, n	Windows opened, n (%)	Windows' adjustment frequency, n	Total no. of AC, n	AC turned on, n (%)	AC adjustment frequency, n	Total no. of fans, n	Fans turned on, n (%)	Fans adjustment frequency, n
H1^A^	39	12 (30.76)	2	261	17 (6.51)	2	0	0 (0)	0	0	0 (0)	0
H2^B^	26	13 (50.00)	4	72	11 (15.27)	4	32	32 (100.00)	2	4	4 (100)	4
H3^B^	10	6 (60.00)	4	16	4 (25.00)	4	4	3 (75.00)	2	16	16 (100)	4
H4^A^	11	6 (54.50)	2	18	0 (0)	2	0	0 (0)	0	0	0 (0)	0
H5^B^	56	21 (37.50)	5	293	80 (27.30)	4	152	150 (98.60)	6	178	178 (100)	2
H6^B^	9	3 (33.33)	4	6	3 (50.00)	6	3	3 (100.00)	2	14	11 (78.57)	5

Note: ^A^ Mechanically ventilated, ^B^ Naturally ventilated.

In terms of the operation of appliances (Table 5), it was mainly observed in OPDs with natural ventilation, as mechanically ventilated OPDs were not equipped with AC and fans. ACs were observed to be fully operated within the OPD unless the AC unit malfunctioned. The frequency of AC adjustment by the OPD occupants was also very minimal, where they mainly turned on the AC in the morning and turned off the AC at the end of the OPD operation in the evening. However, six AC adjustments were observed in H5 OPD during the observational period, as the OPD occupants were trying to cool down the indoor air temperature due to hot weather and high occupancy. Meanwhile, fans were observed to be continuously turned on during the OPD operating hours. Fans were frequently adjusted in small-sized OPDs, such as H3 and H6, as the occupants adjusted the fans' speed according to the indoor air temperature and occupant density at one particular time. In H5, fans were not adjusted throughout the observation period, except for turning the fans on and off at the beginning and end of the OPD operating hours.

### 3.4 Relationship between occupant behaviour and physical IAQ parameters

In terms of the relationship between occupant behaviour and physical IAQ parameters in the selected hospital OPDs (Table 6), occupant density (r = 0.41, p value = 0.01) were positively correlated with indoor air temperature, but not significantly correlated with RH and AV.

**Table 6:** Correlation and R^2^ values between occupant behaviour and physical IAQ parameters in the selected hospital OPDs.

IAQ parameter	Occupant behaviour	Simple correlation^A^	p value	Partial correlation^B^	p value	Correlation by Cohen's classification	R^2^ values^C^	Interpretation
**Temperature**	**Occupant density**	**0.23**	**0.21**	**0.41**	0.01*	**Positive Moderate**	**0.09**	**9 % of temperature variations is explained by occupant density**
	*Activities*							
	Sitting	−0.24	0.17	−0.03	0.88	Negative Weak	0.03	3 % of temperature variations is explained by sitting
	Standing	0.82	<0.001	0.13	0.49	Positive Weak	0.03	3 % of temperature variations is explained by standing
	**Slow walking**	**−0.28**	**0.11**	**0.61**	<0.001***	**Positive Strong**	**0.09**	**9 % of temperature variations is explained by slow walking**
	**Brisk walking**	**0.69**	**<0.001**	**0.47**	0.008**	**Positive Moderate**	**0.10**	**10 % of temperature variations is explained by brisk walking**
	Pushing loaded trolley	0.65	0.02	0.53	0.22	Positive Strong	0.03	3 % of temperature variations is explained by pushing loaded trolley
	Lifting heavy weight	0.01	0.94	0.19	0.31	Positive Weak	0.04	4 % of temperature variations is explained by lifting heavy weight
	*Operation*							
	P**ercentage of doors opened**	**0.36**	**0.03**	**0.63**	<0.001***	**Positive Strong**	**0.14**	**14 % of temperature variations is explained by the percentage of doors opened**
	Doors adjustment frequency	−0.32	0.06	0.35	0.06	Positive Moderate	0.05	5 % of temperature variations is explained by doors adjustment frequency
	**Percentage of windows opened**	**0.77**	**<0.001**	**0.65**	<0.001***	**Positive Strong**	**0.13**	**13 % of temperature variations is explained by the percentage of windows opened**
	Windows adjustment frequency	0.05	0.77	0.03	0.88	Positive Weak	0.07	7 % of temperature variations is explained by windows adjustment frequency
	Percentage of AC turned on	0.06	0.72	0.34	0.06	Positive Moderate	0.06	6 % of temperature variations is explained by the percentage of AC turned on
	AC adjustment frequency	0.79	<0.001	0.27	0.14	Positive Weak	0.05	5 % of temperature variations is explained by AC adjustment frequency
	Percentage of fans turned on	0.81	0.04	0.59	0.21	Positive Strong	0.06	6 % of temperature variations is explained by the percentage of fans turned on
	Fans adjustment frequency	0.79	0.03	0.71	0.15	Positive Strong	0.03	3 % of temperature variations is explained by fans adjustments frequency
**RH**	Occupant density	0.29	0.10	0.14	0.48	Positive weak	0.05	5 % of RH variations is explained by occupant density
	*Activities*							
	Sitting	−0.22	0.21	−0.11	0.66	Negative Weak	0.06	6 % of RH variations is explained by sitting
	Standing	−0.11	0.52	−0.12	0.64	Negative Weak	0.07	7 % of RH variations is explained by standing
	Slow walking	0.28	0.11	−0.31	0.09	Negative Moderate	0.06	6 % of RH variations is explained by slow walking
	Brisk walking	0.21	0.24	0.28	0.12	Positive Weak	0.04	4 % of RH variations is explained by brisk walking
	Pushing loaded trolley	0.13	0.47	−0.02	0.93	Negative Weak	0.05	5 % of RH variations is explained by pushing loaded trolley
	Lifting heavy weight	0.13	0.45	0.08	0.96	Positive Weak	0.03	3 % of RH variations is explained by lifting heavy weight
	*Operation*							
	Percentage of doors opened	0.58	0.52	−0.14	0.44	Negative Weak	0.10	10 % of RH variations is explained by the percentage of doors opened
	Doors adjustment frequency	0.49	0.65	0.47	0.08	Positive Moderate	0.09	9 % of RH variations is explained by doors adjustment frequency
	Percentage of windows opened	0.10	0.60	−0.08	0.67	Negative Weak	0.11	11 % of RH variations is explained by the percentage of windows opened
	Windows adjustment frequency	−0.40	0.02	−0.11	0.66	Negative Weak	0.08	8 % of RH variations is explained by windows adjustment frequency
	Percentage of AC turned on	0.36	0.06	0.32	0.08	Positive Moderate	0.11	11 % of RH variations is explained by the percentage of AC turned on
	AC adjustment frequency	0.14	0.43	−0.20	0.29	Negative Weak	0.05	5 % of RH variations is explained by AC adjustment frequency
	Percentage of fans turned on	0.30	0.47	−0.11	0.54	Negative Weak	0.06	6 % of RH variations is explained by the percentage of fans turned on
	Fans adjustment frequency	0.14	0.44	−0.16	0.38	Negative Weak	0.04	4 % of RH variations is explained by fans adjustment frequency
**AV**	Occupant density	0.22	0.22	0.33	0.5	Positive Moderate	0.05	5 % of AV variations is explained by occupant density
	*Activities*							
	Sitting	0.04	0.08	0.18	0.47	Positive Weak	0.04	4 % of AV variations is explained by sitting
	Standing	0.60	<0.001	0.12	0.51	Positive Weak	0.04	4 % of AV variations is explained by standing
	**Slow walking**	**0.43**	**0.01**	**0.71**	<0.001***	**Positive Strong**	**0.09**	**9 % of AV variations is explained by slow walking**
	**Brisk walking**	**0.61**	**<0.001**	**0.46**	0.009**	**Positive Moderate**	**0.09**	**9 % of AV variations is explained by brisk walking**
	Pushing loaded trolley	0.59	<0.001	0.18	0.31	Positive Weak	0.04	4 % of AV variations is explained by pushing loaded trolley
	Lifting heavy weight	0.08	0.64	0.22	0.29	Positive Weak	0.03	3 % of AV variations is explained by lifting heavy weight
	*Operation*							
	**Percentage of doors opened**	**0.44**	**0.01**	**0.84**	<0.001***	**Positive Strong**	**0.13**	**13 % of AV variations is explained by the percentage of doors opened**
	Doors adjustment frequency	0.20	0.25	0.42	0.08	Positive Moderate	0.06	6 % of AV variations is explained by doors adjustment frequency
	**Percentage of windows opened**	**0.76**	**<0.001**	**0.44**	0.01*	**Positive Moderate**	**0.11**	**11 % of AV variations is explained by the percentage of windows opened**
	Windows adjustment frequency	−0.16	0.37	0.18	0.47	Positive Weak	0.07	7 % of AV variations is explained by windows adjustment frequency
	Percentage of AC turned on	0.06	0.73	0.26	0.16	Positive Weak	0.03	3 % of AV variations is explained by the percentage of AC turned on
	AC adjustment frequency	0.71	<0.001	0.11	0.66	Positive Weak	0.03	3 % of AV variations is explained by AC adjustment frequency
	**Percentage of fans turned on**	**0.68**	**<0.001**	**0.41**	0.02*	**Positive Moderate**	**0.13**	**13 % of AV variations is explained by the percentage of fans turned on**
	Fans adjust frequency	0.71	<0.001	0.31	0.09	Positive Moderate	0.06	6 % of AV variations is explained by fans adjustments frequency

Note: ^A^ Simple correlation analysis: ^B^ Partial correlation analysis, analysis adjusted for age of OPD building, size of OPD, occupant density, type of ventilation system, and ventilation system and appliances maintenance, hygiene, and surveillance history, as well as covariates significant at a p value of 0.25 identified from the simple correlation analyses; ^C^ Multiple linear regression analysis, analysis adjusted for age of OPD building, size of OPD, occupant density, type of ventilation system, and ventilation system and appliances maintenance, hygiene, and surveillance history, as well as covariates significant at a p value of 0.25 identified from simple logistic regression analyses.

*Correlation is significant at < 0.05 level (2-tailed), ** Correlation is significant at < 0.01 level (2-tailed), *** Correlation is significant at < 0.001 level (2 tailed).

Occupant behaviour such as slow walking (r = 0.61, p value < 0.001), brisk walking (r = 0.47, p value = 0.008), percentage of doors opened (r = 0.63, p value < 0.001) and percentage of windows opened (r = 0.65, p value < 0.001) were also positively correlated with indoor air temperature. In relation to indoor air temperature, occupant density (R2 = 9 %), slow walking (R2 = 9 %), brisk walking (R2 = 10 %), and percentage of doors (R2 = 14 %) and windows opened (R2 = 13 %) accounted for the highest indoor air temperature variation.

Meanwhile, slow walking (r = 0.71, p value < 0.001), brisk walking (r = 0.46, p value = 0.009), percentage of doors opened (r = 0.84, p value < 0.001), percentage of windows opened (r = 0.44, p value = 0.01), and percentage of fans turned on (r = 0.41, p value = 0.02) were positively correlated with indoor AV. In relation to indoor AV, slow walking (R2 = 9 %), brisk walking (R2 = 9 %), percentage of doors (R2 = 13 %) and windows opened (R2 = 11 %), and percentage of fans turned on (R2 = 13 %) explained most of the indoor AV variation.

### 3.5 Relationship between occupant behaviour and ventilation performance indicators

In relation to the relationship between occupant behaviour and ventilation performance indicator in the selected hospital OPDs (Table 7), occupant density (r = 0.43, p value = 0.01), slow walking (r = 0.60, p value < 0.001), brisk walking (r = 0.67, p value < 0.001), and percentage of AC turned on (r = 0.45, p value = 0.01) were positively correlated with indoor air CO_2_ levels. On the other hand, the percentage of doors opened (r = −0.55, p value = 0.02) and the percentage of windows opened (r = −0.41, p value = 0.02) were negatively correlated with indoor air CO_2_ levels. Most of the variation in indoor CO_2_ levels was explained by slow walking (R2 = 11 %), brisk walking (R2 = 9 %), percentage of doors opened (R2 = 11 %), percentage of windows opened (R2 = 9 %), and percentage of AC turned on (R2 = 13 %).

**Table 7:** Correlation and R^2^ values between occupant behaviour and ventilation performance indicator in the selected hospital OPDs.

IAQ parameter	Occupant behaviour	Simple correlation^A^	p value	Partial correlation^B^	p value	Correlation by Cohen's classification	R^2^ values^C^	Interpretation
**CO**_**2**_	**Occupant density**	**0.47**	**0.006**	**0.43**	0.01*	**Positive Moderate**	**0.09**	**9 % of CO_2_ variations is explained by occupant density**
	*Activities*							
	Sitting	0.19	0.28	0.12	0.64	Positive Weak	0.04	4 % of CO_2_ variations is explained by sitting
	Standing	0.18	0.32	0.15	0.42	Positive Weak	0.03	3 % of CO_2_ variations is explained by standing
	**Slow walking**	**0.47**	**0.006**	**0.60**	<0.001***	**Positive Strong**	**0.11**	**11 % of CO_2_ variations is explained by slow walking**
	**Brisk walking**	**0.44**	**0.01**	**0.67**	<0.001***	**Positive Strong**	**0.09**	**9 % of CO_2_ variations is explained by brisk walking**
	Pushing loaded trolley	0.38	0.02	0.11	0.53	Positive Weak	0.02	2 % of CO_2_ variations is explained by pushing loaded trolley
	Lifting heavy weight	0.41	0.01	0.35	0.17	Positive Moderate	0.03	3 % of CO_2_ variations is explained by lifting heavy weight
	*Operation*							
	**Percentage ofdoors opened**	**−0.52**	**0.04**	**−0.55**	0.02*	**Negative Strong**	**0.11**	**11 % of CO**_**2**_**variations is explained by the percentage of doors opened**
	Doors adjustment frequency	−0.24	0.17	−0.10	0.60	Negative Weak	0.06	6 % of CO_2_ variations is explained by doors adjustment frequency
	**Percentage of windows opened**	**−0.48**	**0.005**	**−0.41**	0.02*	**Negative Moderate**	**0.09**	**9 % of CO**_**2**_**variations is explained by the percentage of windows opened**
	Windows adjustment frequency	−0.06	0.72	−0.13	0.50	Negative Weak	0.06	6 % of CO_2_ variations is explained by windows adjustment frequency
	**Percentage of AC turned on**	**0.20**	**<0.001**	**0.45**	0.01*	**Positive Moderate**	**0.13**	**13 % of CO_2_ variations is explained by the percentage of AC turned on**
	AC adjustment frequency	0.27	0.12	−0.14	0.45	Negative Weak	0.04	4 % of CO_2_ variations is explained by AC adjustment frequency
	Percentage of fans turned on	−0.46	0.08	−0.49	0.06	Negative Moderate	0.06	6 % of CO_2_ variations is explained by the percentage of fans turned on
	Fans adjustment frequency	0.31	0.08	0.03	0.84	Positive Weak	0.04	4 % of CO_2_ variations is explained by fans adjustment frequency

Note: ^A^ Simple correlation analysis; ^B^ Partial correlation analysis, analysis adjusted for age of OPD building, size of OPD, occupant density, type of ventilation system, and ventilation system and appliances maintenance, hygiene, and surveillance, as well as covariates significant at a p value of 0.25 identified from the simple correlation analyses; ^C^ Multiple linear regression analysis, analysis adjusted for age of OPD building, size of OPD, occupant density, type of ventilation system, and ventilation system and appliances maintenance, hygiene, and surveillance history, as well as covariates significant at a p value of 0.25 identified from simple linear regression analyses. *Correlation is significant at < 0.05 level (2-tailed), ** Correlation is significant at < 0.01 level (2-tailed), *** Correlation is significant at < 0.001 level (2 tailed).

### 3.6 Relationship between occupant behaviour and biological contaminant parameters

In terms of the relationship between occupant behaviour and biological contaminant parameters in the selected public hospital OPDs (Table 8), occupant density was not significantly correlated with TBC (r = 0.21, p value 0.31) and TFC (r = 0.32, p value 0.36).

**Table 8:** Correlation and R^2^ values between occupant behaviour and biological contaminants in the selected hospital OPDs.

IAQ parameter	Occupant behaviour	Simple correlation^A^	p value	Partial correlation^B^	p value	Correlation by Cohen's classification	R^2^ values^C^	Interpretation
**TBC**	Occupant density	0.36	0.04	0.21	0.31	Positive Weak	0.04	4 % of TBC variations is explained by occupant density
	*Activities*							
	Sitting	0.04	0.80	0.01	0.96	Positive Weak	0.05	5 % of TBC variations is explained by sitting
	Standing	0.06	0.81	0.04	0.81	Positive Weak	0.03	3 % of TBC variations is explained by standing
	**Slow walking**	**0.46**	**0.008**	**0.74**	<0.001***	**Positive Strong**	**0.12**	**12 % of TBC variations is explained by slow walking**
	**Brisk walking**	**0.40**	**0.02**	**0.70**	<0.001***	**Positive Strong**	**0.12**	**12 % of TBC variations is explained by brisk walking**
	Pushing loaded trolley	0.62	<0.001	0.22	0.22	Positive Weak	0.05	5 % of TBC variations is explained by pushing loaded trolley
	Lifting heavy weight	0.64	<0.001	0.29	0.11	Positive Weak	0.03	3 % of TBC variations is explained by lifting heavy weight
	*Operation*							
	**Percentage of doors opened**	**−0.75**	**0.001**	**−0.54**	0.005**	**Negative Strong**	**0.11**	**11 % of TBC variations is explained by the percentage of doors opened**
	Doors adjustment frequency	−0.15	0.39	0.24	0.20	Positive Weak	0.04	4 % of TBC variations is explained by doors adjustment frequency
	**Percentage of windows opened**	**−0.51**	**0.002**	**−0.79**	<0.001***	**Negative Strong**	**0.13**	**13 % of TBC variations is explained by the percentage of windows opened**
	Windows adjustment frequency	−0.33	0.05	−0.17	0.37	Negative Weak	0.05	5 % of TBC variations is explained by windows adjustment frequency
	Percentage of AC turned on	0.16	0.35	−0.06	0.75	Negative Weak	0.04	4 % of TBC variations is explained by the percentage of AC turned on
	AC adjustment frequency	−0.49	0.004	−0.11	0.53	Negative Weak	0.03	3 % of TBC variations is explained by AC adjustment frequency
	**Percentage of fans turned on**	**−0.64**	**<0.001**	**−0.43**	0.01*	**Negative Moderate**	**0.10**	**10 % of TBC variations is explained by the percentage of fans turned on**
	Fans adjustment frequency	−0.54	0.001	−0.27	0.15	Negative Weak	0.06	6 % of TBC variations is explained by fans adjustments frequency
TFC	Occupant density	0.24	0.17	0.32	0.36	Positive Moderate	0.03	3 % of TFC variations is explained by occupant density
	*Activities*							
	Sitting	−0.04	0.82	−0.01	0.95	Negative Weak	0.06	6 % of TFC variations is explained by sitting
	Standing	0.39	0.02	−0.12	0.50	Negative Weak	0.07	7 % of TFC variations is explained by standing
	**Slow walking**	**0.32**	**0.07**	**0.57**	<0.001***	**Positive Strong**	**0.09**	**9 % of TFC variations is explained by slow walking**
	**Brisk walking**	**0.17**	**0.35**	**0.54**	0.002**	**Positive Strong**	**0.11**	**11 % of TFC variations is explained by brisk walking**
	Pushing loaded trolley	0.58	<0.001	0.06	0.71	Positive Weak	0.05	5 % of TFC variations is explained by pushing loaded trolley
	Lifting heavy weight	0.60	<0.001	0.11	0.54	Positive Weak	0.05	5 % of TFC variations is explained by lifting heavy weight
	*Operation*							
	**Percentage of doors opened**	**−0.64**	**0.01**	**−0.52**	0.04*	**Negative Strong**	**0.11**	**11 % of TFC variations is explained by the percentage of doors opened**
	Doors adjustment frequency	−0.12	0.50	−0.25	0.15	Negative Weak	0.06	6 % of TFC variations is explained by doors adjustment frequency
	**Percentage of windows opened**	**−0.37**	**0.03**	**−0.63**	<0.001***	**Negative Strong**	**0.11**	**11 % of TFC variations is explained by the percentage of windows opened**
	Windows adjustment frequency	−0.43	0.01	−0.18	0.47	Negative Weak	0.03	3 % of TFC variations is explained by windows adjustment frequency
	Percentage of AC turned on	0.23	0.20	0.23	0.20	Positive Weak	0.07	7 % of TFC variations is explained by the percentage of AC turned on
	AC adjustment frequency	−0.51	0.003	−0.09	0.96	Negative Weak	0.07	7 % of TFC variations is explained by AC adjustment frequency
	Percentage of fans turned on	−0.52	0.002	−0.18	0.35	Negative Weak	0.05	5 % of TFC variations is explained by the percentage of fans turned on
	Fans adjustment frequency	−0.54	0.001	−0.19	0.64	Negative Weak	0.04	4 % of TFC variations is explained by fans adjustments frequency

Note: ^A^ Simple correlation analysis; ^B^ Partial correlation analysis, analysis adjusted for age of OPD building, size of OPD, occupant density, type of ventilation system, and ventilation system and appliances maintenance, hygiene, and surveillance history, as well as covariates significant at a p value of 0.25 identified from the simple correlation analyses; ^C^ Multiple linear regression analysis, analysis adjusted for age of OPD building, size of OPD, occupant density, type of ventilation system, and ventilation system and appliances maintenance, hygiene, and surveillance history, as well as covariates significant at a p value of 0.25 identified from the simple logistic regression analyses.

*Correlation is significant at < 0.05 level (2-tailed), ** Correlation is significant at < 0.01 level (2-tailed), *** Correlation is significant at < 0.001 level (2 tailed).

Meanwhile, occupant behaviour such as slow walking (r = 0.74, p value < 0.001) and brisk walking (r = 0.70, p value < 0.001) were significantly positively correlated with TBC, whereas the percentage of doors opened (r = −0.54, p value = 0.005), percentage of windows opened (r = −0.79, p value < 0.001), and percentage of fans switched on (r = −0.43, p value = 0.01) were negatively correlated with TBC. In relation to TBC, slow walking (R2 = 12 %), brisk walking (R2 = 12 %), percentage of doors opened (R2 = 11 %), percentage of windows opened (R2 = 13 %), and percentage of fans turned on (R2 = 10 %) explained most of the TBC level variation in the selected hospital OPDs.

Slow walking (r = 0.57, p value < 0.001) and brisk walking (r = 0.54, p value = 0.002) were also positively correlated with TFC. On the other hand, the percentage of doors opened (r = −0.52, p value = 0.04) and the percentage of windows opened (r = −0.63, p value < 0.001) were negatively correlated with TFC. In relation to TFC, most of the variations in TFC were explained by slow walking (R2 = 9 %), brisk walking (R2 = 11 %), the percentage of doors opened (R2 = 11 %), and the percentage of windows opened (R2 = 11 %)**.**

### 3.7 Sensitivity analysis and interactions

No significant interaction terms were identified. Sensitivity analyses showed no differences in any outcome and that the study findings are consistent with those from the primary analysis.

## 4 Discussion

The main objective of this study was to examine the relationship between occupant behaviour and IAQ in Malaysian hospital OPDs. Based on the IAQ findings of six randomly selected Malaysian public hospital OPDs and observations of 151 HCWs working in the selected OPDs, after adjusting for potential confounding variables, occupant behaviour including occupant density, occupant activities (slow walking and brisk walking), and occupants' operation of building envelopes and appliances (opening of doors and windows and turning on of fans and AC) were significantly correlated with IAQ parameters. These factors accounted for 67 % of the variation in indoor air temperature, 63 % of the variation in indoor AV, 68 % of the variation in indoor air CO_2_ levels, 66 % of the variation in TBC, and 57 % of the variation in TFC. Among occupants’ behaviour, opening of windows and doors contributed the most to variation in temperature, AV, CO_2_, TBC and TFC.

In this study, the mean IAQ parameter values within the selected hospital OPDs were observed to be in accordance with the ICOP 2010 and ASHRAE standards, except for indoor air temperature and AV. This is similar to the findings of several studies conducted in Malaysian healthcare facilities [73,74], where similar parameters were substandard according to the standard limits. As observed in this study, occupants in naturally ventilated OPDs tended to open windows and doors, whereas those in mechanically ventilated OPDs tended not to. This may have led to a greater influx of warmer outdoor air to flow into the naturally ventilated OPDs’ indoor environment, resulting in higher indoor air temperature and AV, which was not the case for mechanically ventilated OPDs [75]. Though the study findings suggest that the IAQ of Malaysian hospital OPDs is fairly adequate, increased efforts to improve the standard of IAQ in Malaysian hospitals should be made a priority, especially with regard to indoor air temperature and AV. This is because previous studies have shown that indoor air temperature significantly impacts the transmission and viability of microorganisms [76], in which cold ambient temperature, specifically at 4 °C to 6 °C, enhances the stability, viability and survivability of microorganisms including bacteria, fungi, and viruses [77]. Therefore, attaining and sustaining optimal indoor air temperature is important as the vulnerability of patients to HAIs is contingent upon the ability of these germs to survive on different surfaces [77,78]**.** Moreover, insufficient air flow has been shown to cause a build-up of harmful substances in indoor air, including chemicals such as formaldehyde, carbon monoxide, and asbestos, as well as the accumulation of biological substances such as mould spores and other disease-causing microorganisms that thrive in such environments [79]. Therefore, it is equally crucial to ensure optimal indoor AV in hospital indoor spaces.

Occupant density was observed to be positively correlated with two IAQ parameters, i.e., indoor air temperature and CO_2_ levels, and accounted for 9 % of the variations in indoor air temperature and CO_2_ levels. In terms of indoor air temperature, the strong positive correlation between occupant density and indoor air temperature observed in this study is in line with the findings of previous studies [66,80,81]. According to these studies, a significant number of occupants can lead to a notable rise in the indoor air temperature or cooling demand due to the heat emitted by the occupants [80,81]. Additionally, it has been demonstrated that the indoor air temperature decreases rapidly once the indoor space area is emptied [82]. With regard to this, block appointment systems have been acknowledged as an effective strategy in public hospitals to manage patient flow while reducing patient waiting time and waiting room congestion [83]. However, the efficacy of a block appointment system relies on the punctuality of treating doctors, which may not be the case in real situations [84]. The healthcare sector in Malaysia, as in many other nations, is facing increasing demand as a result of factors including an aging population [85], changes in demographic statistics and disease trends, continuous arrival of new immigrants [86], and a surge in medical tourism demand [87], which results in extended waiting periods for outpatient care [88]. Therefore, strategies such as enhancing the OPD block appointment system using time-series extrapolation forecasts and web-based appointments [89] may be potential policy solutions to optimize occupant density and regulate hospital indoor air temperature.

In terms of indoor air CO_2_ levels, the findings of this study are similar to those of previous studies [66], which found that the occupant density of a public building was positively correlated with the indoor air CO_2_ levels. In this regard, building occupants have been shown to contribute to bioeffluent emissions into indoor air, including CO_2_ [66], which is mainly emitted through human breathing. As indoor CO_2_ level is commonly used as a proxy for assessing IAQ and determining appropriate ventilation rates within a building [90], this finding suggests that the current ventilation strategies in Malaysian hospital OPDs with the current occupancy level have a suboptimal effect on reducing indoor air pollutants within the OPD building. Therefore, efforts to improve ventilation and manage indoor air CO_2_ levels within a building space should include controlling the number of building occupants and enhancing the availability of fresh air supplies within the room [66], which can be done in various ways. First, simple actions such as opening doors [91] and windows [92] will allow fresh air into a building with natural ventilation. This method may work effectively in temperate conditions with good outdoor air quality [93]. Second, hybrid ventilation that uses sensors and controllers to determine whether to supply fresh air naturally or mechanically [94] and demand-controlled ventilation (DCV) that utilises sensors in DCV systems to monitor occupancy and air quality in order to adjust fresh air supply to demand [95] may be ventilation systems options to consider given adequate resources.

In this study, occupants' light-intensity and moderate-intensity activities, such as slow walking and brisk walking, were moderately or strongly positively correlated with indoor air temperature, AV, CO_2_, TBC, and TFC. This is in line with findings reported by previous studies, which demonstrated that human activity impacts indoor air temperature and AV [96,97] and that higher intensity activities cause higher levels of indoor air pollutants including CO_2_ and bioaerosols [98]. This is because the human body generates more heat in dynamic conditions compared to static conditions such as sitting or standing [99], whereas human motions have been demonstrated to increase airflow, particularly near and behind the person, which is induced by the moving body and swinging of arms and legs [17,36]. According to these studies, the acceleration of indoor AVs has also been shown to contribute to pollutant distribution in hospital areas. Finally, in relation to indoor CO_2_ and biological contaminant levels, the levels of CO_2_ and bioaerosols are mainly generated by human metabolism and activities [90,100], where higher activity intensity demands that a person breathe quicker thus releasing more CO_2_ and bioaerosols into the indoor air [101]. Given that these IAQ parameters can influence the spread of infectious diseases and cause ill health, measures to regulate occupants’ activities should also be a focus for hospital policymakers and management. Practical approaches for this may include developing IAQ best practice guidelines for HCWs in healthcare settings that incorporate standard procedures for the opening of doors and windows when performing various physical activities in the OPD to facilitate ventilation and remove indoor air contaminants [22]. In addition, given adequate resources, an advanced occupancy sensor system to detect high-intensity activity in hospital indoor areas and trigger ventilation systems to increase air exchange rates may ensure continuous adequate air circulation [102].

The findings of this study also demonstrated that occupants' operation of doors, windows, and fans was strongly or moderately positively correlated with indoor air temperature and AV and strongly or moderately negatively correlated with indoor air CO_2_, TBC, and TFC levels. Conversely, the operation of AC was moderately positively correlated with indoor air CO_2_. This is similar to the findings of previous studies [[103], [104], [105], [106], [107]]. With regard to indoor air temperature, Malaysia is a hot tropical climate country with an average temperature ranging from 26 °C to 30 °C. Thus, the occupants' behaviour of opening and adjusting windows and doors in the selected public hospital OPDs would allow a significant amount of warmer outdoor air to flow into the OPD indoor environment, thus increasing the indoor air temperature [75]. In relation to indoor AV, manipulating doors, windows, and appliances can increase the AV indoors, as opening a door or window creates a pathway through which air can freely move between indoors and outdoors [94,105]. Furthermore, appliances such as fans or ACs are intentionally designed to optimise AV by producing a stream of air or redistributing cooled air throughout the surrounding area [108]. Indeed, previous studies have indicated that building occupants often open their windows to adjust the indoor air temperature and improve airflow [42] and utilise fans to provide faster air movement and a cooling effect induced under slightly warm conditions [109]. Concerning indoor air CO_2_, TBC, and TFC levels, the findings of this study are also in line with previous studies, which have demonstrated that the opening of doors and windows results in indoor air replacement with outdoor air, which reduces indoor air contaminants, including CO_2_, TBC, and TFC [107,110,111]. Meanwhile, the operation of fans in the hospital OPDs induces air currents indoors [112], which, when coupled with the opening of doors and windows, promote indoor-outdoor air exchange [113] and dispersion of suspended bioaerosols to the outdoors [112], thus reducing the bioaerosol concentration indoors. Nevertheless, from the observation, even though hospital OPD occupants are free to adjust fan speeds during their time spent in the OPDs, many still do not optimsie the fan operations in the OPDs. Practical measures to ensure occupants’ best practice of operating building envelopes and fans to optimise indoor air temperature, AV, and control indoor contaminants may include the following. Firstly, applying adjustable blinds, drapes, and shades on windows facing direct sunlight would enable occupants to open windows and allow good airflow and air exchange yet maintain optimal indoor air temperature [114]. Secondly, besides windows, doors can also be opened for 20 minutes every 4 hours to introduce fresh air into the indoor environment and dilute the indoor air contaminant concentrations [115]. Thirdly, during the performance of strenuous activities in the OPDs, HCWs should keep the doors and windows open to reduce any build-up of indoor air contaminants. Finally, HCWs should maximise the fan speed to enhance indoor airflow, lower indoor air temperature, and improve thermal comfort during hot sunny days [116,117].

On the other hand, the recirculation process by AC can result in the accumulation of CO_2_ indoors due to several factors, including restricted ventilation, high occupancy, and airtight building envelopes [110,118]. Restricted ventilation in indoor environments can lead to a progressive elevation of CO_2_ levels due to the exhalation of CO_2_ by occupants during the process of respiration [110]. Additionally, during the operation of the air conditioning system, individuals tend to maintain the desired temperature by keeping doors and windows closed, which restricts the influx of fresh air [110]. In this study, the selected public hospital OPDs were all designed to have enclosed rooms with ACs for doctor‒patient consultation sessions and various medical procedures to ensure confidentiality and privacy. Therefore, practical solutions to reduce the build-up of indoor CO_2_ levels in areas with AC usage may be to introduce regular intermittent openings of building envelopes such as doors and windows to allow outdoor fresh air to flow into naturally ventilated indoor areas installed with AC units [119]. HCWs should also optimally operate and manipulate the AC to ensure that the indoor air temperature is always within the standard range, i.e. 23 °C to 26 °C.

This study has several limitations that should be acknowledged. First, the cross-sectional nature of the study design does not permit inferences to be made regarding causality. Second, as this study was conducted in Malaysian public hospital OPDs, caution should be exercised when generalising the findings to hospital OPDs in other parts of the world, as the hospital setting, infrastructure, occupants' characteristics, and practice may differ [120]. Third, since this study was performed using observational data collection, there was no opportunity to learn occupants' behaviour in terms of their previous activities and operation of doors, windows, and appliances in the selected public hospital OPDs. It was also difficult to gather information on intentions, opinions, attitudes, or preferences during the study data collection. Fourth, this study did not include other behavioural metrics such as patients’ consultations and waiting periods, and interactions between different occupants within the OPD indoor environment. Though this was not performed due to resource limitations, including these behavioural metrics may provide further insights into occupant-related IAQ factors. Fifth, there was a possibility of the Hawthorne effect influencing the findings of this study, as the HCWs in the selected OPDs may have behaved differently from their norm during the observation period. Nonetheless, this may have been minimised as the confidentiality of the study was reassured to the OPD occupants prior to data collection. Sixth, due to research fund limitation, additional IAQ parameters such as PM (PM_10_ and PM_2.5_), volatile organic compounds (VOCs) and ozone were unable to be included in this study. This is unfortunate as these pollutants can also significantly impact IAQ and human health. Finally, residual confounding from non-adjustment of non-available data such as material composition of the OPDs [14] may have altered the true association between factors and the outcome observed in this study.

## 5 Conclusions

Based on the findings of this study, occupant behaviour, including occupant density, activities, and operation of building envelopes and appliances were significant factors of hospital OPD IAQ. Given that these factors are modifiable and amenable to prospective IAQ interventions, the present study provides evidence-based focuses for healthcare policymakers, hospital administrators, environmental specialists, and infection control teams to develop policies and occupant-driven control and mitigation strategies to improve hospital OPD IAQ. Future studies should include additional chemical parameters to ascertain the level of chemical pollutants present in hospital OPD indoor air and impact of occupants' behaviour on these parameters, as well as qualitative studies to further explore occupants' personal and environmental adaptive behaviour in relation to IAQ. Additionally, future researchers may consider including behavioural metrics of other OPD occupants besides HCWs in their study, including specific activities of OPD patients and visitors for a comprehensive assessment of IAQ-related hospital occupant behaviour. In strategising and optimising hospital IAQ, either via mechanical or natural indoor air control systems, we must consider the uncertainty and complexity of occupants’ behaviour, which is mainly based on their varying preferences about the indoor environment and control habits of the system. Therefore, multipronged strategies considering these important occupant-related determinants are deemed a viable strategy for the future control and improvement of hospital IAQ.

## Ethics and consent statement

This study was reviewed and approved by UiTM Research Ethics Committee (UREC) with the approval number: [REC/08/2022 (PG/MR/193], dated August 12, 2022, as well as the Medical Research Ethics Committee (MREC) with the approval number: [NMRR ID-22-00842-VCH (IIR)], dated May 26, 2022. All participants provided written informed consent to participate in the study and for their data to be published.

## Data availability statement

The data that support the findings of this study are available from Universiti Teknologi MARA (UiTM). Restrictions apply to the availability of these data, which were used under license for this study. Data are however available from the authors upon reasonable request and with the permission of UiTM.

## Funding

The Geran Penyelidikan Dana UiTM Cawangan Selangor (DUCS) 4.0 [600-UiTMSEL (PI.5/4) (015/2022)] provided financial support for the conduct of the research. The funder had no role in the study design, collection, analysis, and interpretation of data, writing of report, and decision to submit the article for publication.

## CRediT authorship contribution statement

**Farha Ibrahim:** Writing – original draft, Visualization, Project administration, Methodology, Investigation, Formal analysis, Conceptualization. **Ely Zarina Samsudin:** Writing – review & editing, Visualization, Supervision, Methodology, Funding acquisition, Formal analysis, Conceptualization. **Ahmad Razali Ishak:** Writing – review & editing, Resources, Methodology, Formal analysis. **Jeyanthini Sathasivam:** Writing – review & editing, Resources.

## Declaration of competing interest

The authors declare the following financial interests/personal relationships which may be considered as potential competing interests: Dr. Ely Zarina Samsudin reports administrative support and equipment, drugs, or supplies were provided by Universiti Teknologi MARA Cawangan Selangor. If there are other authors, they declare that they have no known competing financial interests or personal relationships that could have appeared to influence the work reported in this paper.
